# U.S. English‐Speaking Children and Adults Exhibit a “Gleam‐Glum” Sound Symbolic Effect Linking Phonemic Vowel Sounds With Emotional Valence

**DOI:** 10.1111/cogs.70215

**Published:** 2026-04-29

**Authors:** Ye Li, Christine S.‐P. Yu, Michael K. McBeath, Viridiana L. Benitez

**Affiliations:** ^1^ Department of Psychology Arizona State University; ^2^ Department of Language Science and Technology The Hong Kong Polytechnic University; ^3^ Department of Music Max Planck Institute for Empirical Aesthetics

**Keywords:** Sound symbolism, Emotion, Phonemic valence, Language development, Children versus adults

## Abstract

We tested a recently‐found sound symbolic effect, the *gleam‐glum effect*, in which words with the [i]‐phoneme (like “*gleam*”) are perceived as emotionally more positive than matched words with the [Ʌ]‐phoneme (like “*glum*”). We extend prior work and verify this effect using a novel online pseudoword‐to‐scene matching task, testing U.S. English‐speaking adults (*n* = 105) and 5‐ to 7‐year‐old children (*n* = 52). Participants heard pairs of matched [i]‐ versus [Ʌ]‐monosyllabic pseudowords (e.g., “*zeem*” versus “*zum*”) and assigned them to cartoon scenes exhibiting contrasting emotional valence (positive versus negative). These results provide the first empirical evidence that the gleam‐glum effect is robust across both young children and adults, with the effect magnitude somewhat less in children of this age compared to adults. Our findings confirm that the gleam‐glum effect is already strong at an early age and holds promise of being an important mechanism for language comprehension, language use, and language learning.

## 1 Introduction

Using and learning spoken language effectively requires deciphering the meanings of words. Multiple factors can help listeners identify correct word‐to‐world mappings, including associative (Benitez & Saffran, 2018; Suanda, Mugwanya, & Namy, 2014; Yu & Smith, 2007), social (Baldwin, 1993; Bloom, 2000; Tomasello, 1992), and syntactic (Fisher, Gertner, Scott, & Yuan, 2010; Gleitman, 1990) cues. Here, we examine one type of cue that may scaffold adult and child listeners to identify word‐to‐meaning mappings—that phonemes convey emotional valence.

### 1.1 Sound symbolism

Contrary to a traditional view that word‐to‐meaning mappings are arbitrary (de Saussure, 1916), some non‐arbitrary associations between phonemes and their meanings exist, termed sound symbolism (e.g., Blasi, Wichmann, Hammarström, Stadler, & Christiansen, 2016; Lockwood & Dingemanse, 2015; Sidhu & Pexman, 2018). For example, across many spoken natural languages, specific phonemes are associated with shape (Blasi et al., 2016), size (Blasi et al., 2016; Winter & Perlman, 2021), proximity (Johansson & Zlatev, 2013; Rabaglia, Maglio, Krehm, Seok, & Trope, 2016), and texture (Winter, Sóskuthy, Perlman, & Dingemanse, 2022). Additionally, iconic words, words that resemble their meanings in some form, have been found to be acquired earlier and introduced more frequently in infant‐directed‐speech across languages (e.g., Asano et al., 2015; Jo & Ko, 2018; Sidhu, Williamson, Slavova, & Pexman, 2022), supporting that iconicity may aid early word learning (e.g., Imai & Kita, 2014; Laing, 2019; Monaghan, Shillcock, Christiansen, & Kirby, 2014; Nielsen & Dingemanse, 2021; Perry, Custode, Fasano, Gonzalez, & Savy, 2021; Perry, Perlman, & Lupyan, 2015; Thompson, Vinson, Woll, & Vigliocco, 2012). Further, phonemes in novel words can bias learners’ selection of referents (Imai et al., 2015; Kantartzis, Imai, & Kita, 2011; Lupyan & Casasanto, 2015; Monaghan, Christiansen, & Fitneva, 2011; Yoshida, 2012). For example, adults, children, and infants typically link novel pseudowords like “*bouba*” to round shapes and “*kiki*” to spiky shapes, termed the *bouba‐kiki effect* (e.g., Fort et al., 2018; Maurer, Pathman, & Mondloch, 2006). This effect has been shown to increase with age in early childhood, from 3 to 7 years of age (Tzeng, Nygaard, & Namy, 2017). These classic types of sound symbolism challenge the traditional view on the arbitrary link between words and meanings.

Critically, both using spoken language to communicate effectively and the acquisition of spoken language may benefit from the presence of sound symbolism (Lockwood & Dingemase, 2015). To date, studies assessing sound symbolism have largely focused on identifying the link between word sounds and the perceptual properties of their meanings (e.g., shape, size, and texture). Yet evidence has emerged that sound symbolic effects could extend to meanings beyond physical features of referents. In the current study, we provide new evidence supporting that emotion can also serve as a broad type of sound symbolism by testing the *gleam‐glum effect*, using a novel methodology with English‐speaking adults and 5‐ to 7‐year‐old children.

### 1.2 Emotional sound symbolism

Emotion has been proposed to be a critical dimension for organizing meaning in the world and the words that are linked to those meanings (Vigliocco, Meteyard, Andrews, & Kousta, 2009). Language is strongly intertwined with emotion, with many aspects of language proposed to convey emotional features (Majid, 2012). For example, Warriner, Kuperman, and Brysbaert (2013) found that people could provide ratings of valence, arousal, and dominance for a large range of words, with the first two features being principal categories of emotion. These critical features of emotion have likely been part of the experiences shared between humans and non‐human species (Darwin, 1872; Paul, Sher, Tamietto, Winkielman, & Mendl, 2020) and by some accounts, human emotion co‐evolved with language, such that emotional expression could have facilitated more sophisticated forms of communication, paving the way for the origin and evolution of language (e.g., Filippi, 2020; Jablonka, Ginsburg, & Dor, 2012). Given the link between language and emotion, understanding whether and how words convey emotion information may be critical to understanding how sound symbolism can be fundamental to language comprehension, language use, and language learning, as well as possibly even the evolution of human communication.

An emerging body of work has begun to provide evidence for emotional sound symbolism, focusing specifically on how individual phonemes themselves may systematically convey valence information, such as positive or negative affect (e.g., Adelman, Estes, & Cossu, 2018; Aryani, Conrad, Schmidtke, & Jacobs, 2018; Conrad, Ullrich, Schmidtke, & Kotz, 2022; Garrido & Godinho, 2021; Kambara & Umemura, 2021; Rummer & Schweppe, 2019; Rummer, Schweppe, Schlegelmilch, & Grice, 2014; Yu, McBeath, & Glenberg, 2021a, 2021b). Findings indicate that valence information is carried in the phonemes of words across many languages (Adelman et al., 2018). Further, specific phonemes in pseudowords seem to bias adults to rate and link words with positive or negative valence (e.g., Körner & Rummer, 2022; Rummer & Schweppe, 2019; Rummer et al., 2014; Schmidtke, Körner, Glim, & Rummer, 2025; Yu et al., 2021a).

### 1.3 The gleam‐glum effect

A notable example of emotional sound symbolism is the *gleam‐glum effect*, which proposes that words with the [i]‐phoneme are perceived as more emotionally positive and words with the [Ʌ]‐phoneme as more emotionally negative (see Yu et al., 2021a, 2021b).^1^ The gleam‐glum distinction was initially formulated based on multidimensional scaling of perceptual similarity judgments of different phonemes. There it was found that [i] and [Ʌ] were rated as two of the most perceptually dissimilar vowel phonemes in English and two extremes of timbre brightness and spectral centroid (Patten, McBeath, & Baxter, 2019; Patten & McBeath, 2020, 2025). The distance in the perceptual similarity mappings for these two phonemes also largely matches the opposing configuration of [i] and [Ʌ] in maps of vowel phonemes along the first two acoustic formants and orofacial musculature models of speech production (Yu et al., 2021b). Additionally, [i] was rated as the phoneme that sounds highest in pitch and [Ʌ] lowest by English‐speaking participants, both perceptually and in production when speaking and singing (Patten & McBeath, 2020, 2025). Further, higher pitch has been found to be associated with positive valence and lower pitch with negative valence (e.g., Barber & Reimer, 2021; Kamiloğlu, Fischer, & Sauter, 2020). Finally, the [i] phoneme is produced with the same orofacial musculature of a smile, while the [Ʌ] phoneme is produced with musculature similar to a type of frown or grimace. Together, these studies point to [i] and [Ʌ] being contrasting extremes on production and perception along multiple converging dimensions that are likely to be interrelated. Importantly, these distinctions may be related to emotional expression, making the gleam‐glum dichotomy in English a promising candidate for an emotional sound symbolic effect with the [i] phoneme signaling more positive valence and the [Ʌ] phoneme more negative valence (Yu et al., 2021b).

The first empirical tests of the gleam‐glum effect with adults demonstrated evidence supporting the effect. Results confirmed that adults rated real English words with the [i]‐phoneme (like “*peace*”) as more emotionally positive, and matched words with the [Ʌ]‐phoneme (like “*pus*”) as more emotionally negative (Yu et al., 2021a). The gleam‐glum effect was found in 63% of the entire set of all extant monosyllabic English word pairs containing [i] versus [Ʌ], exhibiting a large effect size (Cohen's *d* > 1). In addition, the gleam‐glum effect was also found to be robust for Mandarin Chinese words rated by Mandarin Chinese‐speaking adults (Yu et al., 2021a).

Further studies confirmed the robustness of the gleam‐glum effect by adding neutral controls. Yu et al. (2021b) had adult listeners rate the relative emotional valence of matched single‐syllable word‐pairs that contained the same beginning and ending consonant phonemes but with each word containing a middle vowel phoneme from the perceptual similarity vowel continuum spanning [i] to [Ʌ]. These included the phonemes [i] (as in “*deal*”), [I] (as in “*dill*”), [Ↄ] (as in “*doll*”) and [Ʌ] (as in “*dull*”). Findings showed that words containing the phoneme [i] were rated the most positive, [Ʌ] the most negative, with [I] and [Ↄ] being in between and not statistically different from each other. These findings confirmed that the [i] and [Ʌ] phonemes are, respectively, interpreted as more positive and negative than other more neutral English control phonemes to which they were compared.

### 1.4 The current study

Taken together, the gleam‐glum effect supports the claim that sound symbolism can extend beyond physical properties of a referent to emotional components of words, cueing listeners into the valence of words. However, the link between phonemes and emotional valence has not been previously tested in children. This is a critical gap in the literature for several reasons. First, understanding the pattern of emotional sound symbolism across development can provide insights into mechanisms driving sound symbolic effects (e.g., Imai & Kita, 2014; Sidhu & Pexman, 2018; Tzeng, Nygaard, & Namy, 2017). Additionally, this understanding can help clarify the impact of emotional sound symbolism in language learning, language development, and language use (e.g., Körner & Rummer, 2022; Nielsen & Dingemanse, 2021). Further, despite the strong links between language and emotion, these two domains have largely been examined separately in early development with only a few exceptions (e.g., Bloom & Beckwith, 1989; Nencheva, Tamir, & Lew‐Williams, 2023). Identifying whether young children exhibit biases to link language and emotion information can contribute to a more integrated understanding of the interplay between emotion and language in early childhood. Finally, sound symbolism has been proposed as key to early language learning (see a review in Nielsen & Dingemanse, 2021). The sound symbolism bootstrapping hypothesis (Imai & Kita, 2014) posits that multisensory integration (e.g., mapping the sound of a word with the sensorimotor features of its meaning) narrows down the scope of candidate referents in a scene when hearing a novel word, which scaffolds word learning in ambiguous learning environments, though the scope of such a bootstrapping effect is under evaluation (i.e., Nielsen & Dingemanse, 2021). If the gleam‐glum effect is present in young children, then this provides an important initial step to further test whether (and how) such a sensitivity to the sound–emotion associations early on may scaffold language learning. In contrast, if the gleam‐glum effect is weak or absent in young children, this would favor that the effect requires long‐term experience with language and may play a peripheral role in early language learning. The *first aim* of this study is to develop a novel and child‐friendly methodology that allows us to test the gleam‐glum effect in both adults and young children. This will also provide the first test of the gleam‐glum effect in young children.

A *second aim* is to compare the gleam‐glum effect between young children and adults, as this provides further insights into how the mechanisms underlying the effect change across development. One possibility is that this sound symbolic effect is more robust among adults, compared to young children: for instance, the extant English lexicon predominantly obeys the rules of the gleam‐glum effect (Yu et al., 2021a), and long‐term language experience with English may strengthen this link. Another possibility is that the gleam‐glum effect is not different in young children compared to adults. This would suggest that the gleam‐glum effect is stable and not subject to the effects of long‐term language experience.

In this study, we provide a first test of the gleam‐glum effect in English‐speaking 5‐ to 7‐year‐old children and compare this effect to English‐speaking college students using a novel online pseudoword‐to‐scene matching task. We chose 5‐ to 7‐year‐olds as the first test of the gleam‐glum effect in children for several reasons. First, this age group can process the emotional valence of complex scenes (Theurel et al., 2016), which was necessary to complete our pseudoword‐to‐scene matching task. Second, although this age group has developed complex comprehension and production skills for language and emotion, they are still making significant advances in learning vocabulary (Bloom, 2000) and honing their emotion processing skills (Declercq, Marlé, & Pochon, 2019; Pons, Harris, & De Rosnay, 2004). Finding the effect in this age group is an important first step toward identifying whether young children adopt emotional sound symbolism for comprehending, using, and learning language, as well as for motivating testing the effect at even younger ages.

## 2 Method

The study was pre‐registered on the Open Science Framework (OSF) for children (https://doi.org/10.17605/OSF.IO/TU7J3) and adults (https://doi.org/10.17605/OSF.IO/AZQXU). Both children and adults completed a pseudoword‐to‐scene matching task in which on each trial, two cartoon scenes were presented side‐by‐side. The two scenes displayed the same cartoon character but contrasted in emotional valence (one positive and the other one negative). Participants then heard two aurally presented novel pseudowords that differed only in their vowel phoneme, one containing the [i]‐phoneme and the other containing the [Ʌ]‐phoneme. Participants were then asked to map the referent of one of the two pseudowords onto their choice of one of the two scenes.

### 2.1 . Participants

#### 2.1.1 Children

Children aged 5‐ to 7‐years (*n* = 52, *M_age_* = 6.45, age range: 5.08–7.75, 27 female and 25 male) and their parents were recruited from the Phoenix Metropolitan area and online. Local recruitment utilized flyers distributed at preschools and the Children's Museum of Phoenix. Online recruitment involved social media posts on various platforms. Child participants were predominantly from the Phoenix Metropolitan area (*n* = 29), while some were from regions elsewhere in Arizona (*n* = 3) and from other states in the United States (*n* = 17), with three missing location data. Parents and their children participated online via a scheduled Zoom session. Parents completed a demographic and language questionnaire for their children after the study (see Table 1).

**Table 1:** Participants’ demographic information

	Children	Adults
Age		
Mean (*SD*)	6.45 (.80)	19.18 (1.41)
Range	5–7.75	18–25
Gender (%)		
Female	27 (51.9%)	39 (37.1%)
Male	25 (48.1%)	66 (62.9%)
Language (%)		
Speak English only	40 (76.9%)	65 (61.9%)
Speak English and other language(s)	10 (19.2%)	40 (38.1%)
Race/ethnicity (%)		
White	26 (50.0%)	44 (41.9%)
American Indian or Alaska Native	0 (0.0%)	3 (2.9%)
Black or African American	0 (0.0%)	8 (7.6%)
Asian	8 (7.6%)	20 (19.0%)
Hispanic or Latino	5 (4.8%)	18 (17.1%)
Multiple	12 (11.4%)	9 (8.6%)
Other	1 (1.9%)	3 (2.9%)
Parental education (%)		
Some high school	0 (0.0%)	7 (6.7%)
High school/General educational development	0 (0.0%)	25 (23.8%)
Some college or associates	6 (11.5%)	38 (36.2%)
Bachelors	20 (38.5%)	27 (25.7%)
Advanced education (masters or PhD)	26 (50.0%)	8 (7.6%)
Total number	52	105

*Note*. For Age: Mean, each cell represents the mean age per age group (standard deviation in parenthesis). For Age: Range, each cell denotes the minimum and the maximum number of ages per age group. For Gender, Language, Race/Ethnicity, and Parental education (the higher education from both parents), each cell represents the number of participants per age group (percentage in parentheses). For Language, there are two missing data for the Children group (3.8%).

Data from children with parental‐reported developmental impairments (*n* = 3) and who failed to complete the task (*n* = 3) were excluded. Consent and assent procedures were conducted in accordance with the Arizona State University (ASU) Institutional Review Board (IRB). Parents consented to their child participating in the study. Children younger than 7 additionally gave verbal assent, while children aged 7 years and older additionally provided written assent. Families received a $10 e‐gift card and an electronic coloring book for their participation.

#### 2.1.2 Adults

English‐speaking college students (*n* = 105, *M_age_* = 19.18, age range: 18–25, 39 female and 66 male) were recruited from the ASU Introduction to Psychology subject pool. Adult participants were given an online link to self‐administer the task and asked to fill out a demographic and language background questionnaire afterwards (see Table 1). Forty adults reported knowing a language in addition to English. One additional adult was excluded due to no recorded responses. Adults received course credit to complete the study. Consent was provided according to the ASU IRB.

### 2.2 Stimuli

English‐like monosyllabic pseudowords were constructed in yoked pairs sharing the same consonant‐vowel‐consonant (CVC) word frame (e.g., gl __ p gives “*gl* ***ea*** *p*” and “*gl* ***u*** *p*”). Specifically, we found all yoked pairs of spelling bodies (i.e., the vowel and ending consonant of the word – “*eap*” and “*up*” in the current example) in English that included the [i] and [Λ] vowel sounds (Ziegler, Stone, & Jacobs, 1997). We used only spelling bodies that were feed‐forward consistent, meaning that the spelling bodies had only one possible pronunciation (Stone, Vanhoy, & Van Orden, 1997). This was done to maximize the likelihood that researchers recording the words pronounced the pseudowords as intended. We then semi‐randomly attached initial consonants to the spelling bodies to produce the list of CVC pseudowords. Finally, we eliminated pseudowords with any possible real‐world meanings (i.e., pseudo‐homophones such as “*keap*” and slang such as “*yeet*”). This resulted in 50 yoked pairs of monosyllabic CVC pseudowords that were identical in pronunciation except for the vowel at the mid position and that have been used in a prior study demonstrating the gleam‐glum effect in adults (Yu et al., 2021a). For this study, we selected 32 pairs to create a version of the task suitable for children's shorter attention span. We selected the most distinctive 32 pairs by eliminating among pairs that were phonologically similar (e.g., “*smeem*‐*smum*” was eliminated because of its similarity with “*smeen*‐*smun*” and “*sneme*‐*snum*”). Table 2 displays all 32 pairs of pseudowords.

**Table 2:** Pseudoword pairs used in the study

[i]‐Pseudoword	[Λ]‐Pseudoword
*bleem*	*blum*
*breap*	*brup*
*dreek*	*druck*
*dreen*	*drun*
*cleem*	*clum*
*crene*	*crun*
*fleech*	*fluch*
*freap*	*frup*
*freen*	*frun*
*gleap*	*glup*
*greech*	*gruch*
*keach*	*kuch*
*pleech*	*pluch*
*plene*	*plun*
*preep*	*prup*
*scheach*	*schuch*
*screep*	*scrup*
*sleech*	*sluch*
*smeach*	*smuch*
*smeap*	*smup*
*smeen*	*smun*
*sneme*	*snum*
*spleek*	*spluck*
*spleem*	*splum*
*spream*	*sprum*
*spreek*	*spruck*
*streech*	*struch*
*threne*	*thrun*
*treach*	*truch*
*yeach*	*yuch*
*zeek*	*zuck*
*zeem*	*zum*

*Note*. The table displays the 32 pseudoword pairs used in the study. Each line contains a pseudoword pair. In each pair, the left column lists the pseudoword containing the [i] vowel, and the right column lists the corresponding pseudoword containing the [Λ] vowel. The pseudoword pairs are listed in alphabetical order according to the [i]‐pseudoword in a pair.

The final 32 pairs of pseudowords were recorded in child‐directed speech by a female U.S. English‐speaking research assistant who was unaware of the study hypothesis. An individual audio file was created for each pseudoword. We explored whether the [i]‐ and [Ʌ]‐pseudoword recordings differed in pitch. Results showed that [i]‐ and [Ʌ]‐pseudowords did not differ significantly in pitch^2^.

The researcher additionally recorded four carrier phrases (e.g., “Here I have,” “And there is also”) as well as four test phrases (e.g., “Can you choose”). This allowed us to pseudorandomly combine pseudoword pairs, carrier phrases, and test phrases. As for the duration of individual items, pseudowords (0.80 s), carrier phrases (1.50 s), and test phrases (1.50 s) were modified to be the same for each category. All auditory stimuli can be found on OSF (https://osf.io/yqd6g/).

The scene stimuli were created by an artist without knowing the study hypothesis. The artist was asked to create scenes that depicted cartoon animals in situations that young children could understand. The artist was instructed to delineate each cartoon animal in complementary emotionally positive and negative scenes, the scenes being as similar as possible except for emotional valence. In other words, the differences between a pair of contrasting scenes signaled the emotional valence of each scene. All visual stimuli can be found on OSF (https://osf.io/yqd6g/). We asked a separate group of college students (not included in this study; *n* = 28) to rate a set of pairs of scenes for their emotional valence. We selected our final scene stimuli to include those 32 pairs of scenes with the most salient emotional valence contrast.

### 2.3 Design

**Fig. 1:**
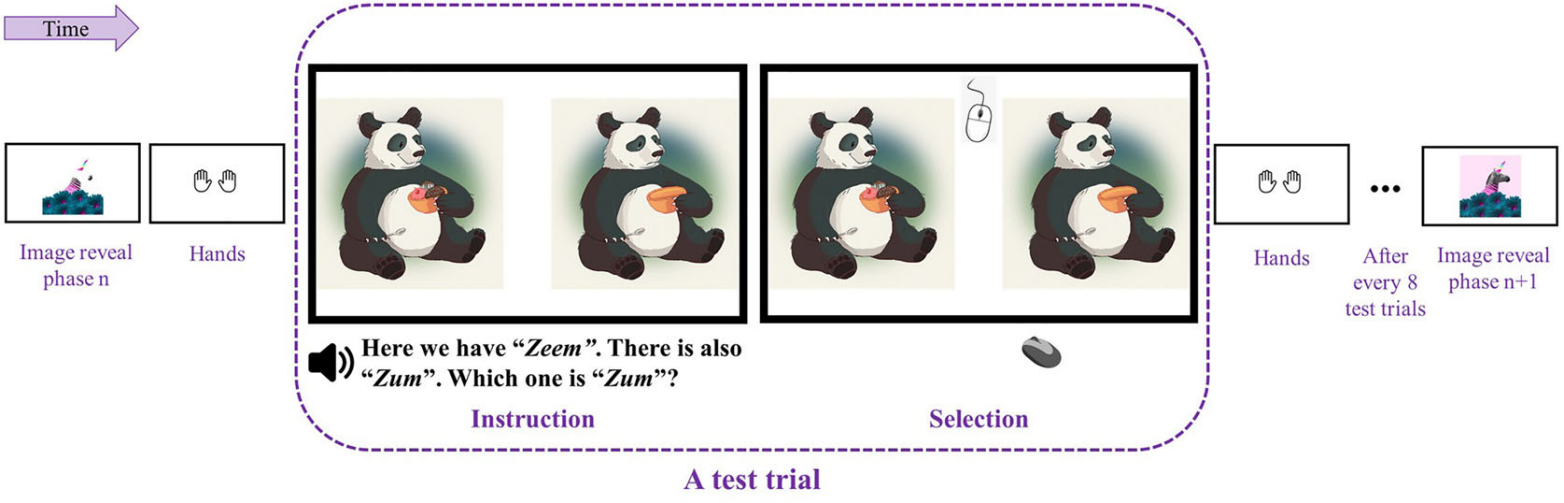
Example test trial in the pseudoword‐to‐scene mapping task (children and adults). Each test trial started with instructions where participants heard both an [i]‐phonemic monosyllabic pseudoword (e.g., “*zeem*”) and its matched [Ʌ]‐phonemic counterpart (e.g., “*zum*”) and saw two scenes with contrasting emotional valence (e.g., a happy panda and a sad panda). Then, during selection, participants were asked to click on the scene that best matches a specified test pseudoword (“*zum*” in this case). Both children and adults completed a total of 32 randomized test trials. To ensure engagement during the task, children additionally saw a pair of safety hands before each test trial (Hands) and a series of images incrementally revealing a scene after every 8 test trials (Image reveal phase *n* and phase *n* + 1.)

In the pseudoword‐to‐scene mapping task, all participants completed 32 test trials. Each test trial started with an instruction (Fig. 1. Instruction), presenting a pair of scenes in which a cartoon character demonstrated contrasting valence of being emotionally positive and negative (e.g., a happy panda vs. a sad panda). After 1 s, participants were aurally introduced with a pair of [i]‐ versus [Ʌ]‐pseudowords, such as “*zeem*” and “*zum*,” each embedded in a carrier phrase, such as “Here we have *zeem*. There is also *zum*.” Presentation order was randomized for both pseudoword pairs and scene pairs such that half of the trials began with [i]‐pseudowords and the other half with [Ʌ]‐pseudowords and half with positive scenes to the left and the other half to the right. Each test word was embedded in a test phrase, such as “Which one is zum?” with half of the test words being [i]‐pseudowords and the other half being [Ʌ]‐pseudowords. After the instruction, participants were instructed to select the target scene that best matched the chosen test word (Fig. 1. Selection). To counterbalance the spatio‐temporal order of word‐to‐scene mappings, a total of 16 test lists were created and randomly assigned across participants.

### 2.4 Procedure

The task was built using PsychoPy Builder (version 2020.2.10; Peirce et al., 2019) and launched online using Pavlovia (https://pavlovia.org/; Bridges et al., 2020). The PsychoPy program built for this task can be found on OSF (https://osf.io/yqd6g/). Consent and questionnaires were created in Qualtrics (https://www.qualtrics.com).

#### 2.4.1 Children

The parent and their child were scheduled for an online session via the Zoom platform (https://zoom.us/) to complete the study with a live experimenter. A Zoom session allowed for the testing of one child at a time. At the start of the online session, an experimenter explained the study as “The Name Game” where the child would have to guess the names of some pictures. After explaining the procedures of the study, parental consent and verbal or written assent from the child were obtained. The experimenter then shared their screen with the task displayed and gave remote control access of the mouse to the child (a feature on Zoom) so the child could click on the images they were seeing through the shared screen.

Differing from adults, the task for children included some additional steps to ensure their understanding of the instructions and to maintain their engagement. The experimenter first explained the task as a game. Children were then shown a pair of handprints and told that every time they saw this image, they were to place their hands next to the computer (Fig. 1. Hands). Children were then shown a picture of a computer mouse and instructed that when the computer mouse appeared, they could use their own computer mouse to click on an image. This ensured that children would not make a selection before they heard the complete auditory stimuli.

Children were then introduced to a character, Alex, who loved to draw animals and had special names for each of her drawings. Children's goal in the game was to guess the special name of each drawing. Before the actual test trials, children completed two practice trials to become familiarized with the pseudoword‐to‐scene mapping task. The images and novel words shown in the practice trials did not appear in the actual test trials.

After the practice trials, children were presented with an image with most portions hidden (Fig. 1. Image reveal). The image reveal served to increase children's task engagement and would reveal itself incrementally after each of eight test trials (i.e., the image was completely revealed after the entire 32 test trials). A live experimenter instructed children at each image reveal in child‐directed speech (e.g., “Look! What's this?”) to ensure their attention.

After the practice trials and instructions on the image reveal, children completed the actual test trials (Fig. 1. A test trial). The task for children lasted less than 10 min. Parents were asked to fill out a demographic survey during or after the child completed the task and then were debriefed at the end. The entire session lasted 30–45 min for children.

#### 2.4.2 Adults

Undergraduate students were given an online URL link to complete the study on their own. The URL link started with the consent form, written instructions on how to complete the task, followed by the pseudoword‐to‐scene matching task, and finally a demographic and language background questionnaire. For the pseudoword‐to‐scene matching task, adults completed 32 test trials in succession (Instruction‐then‐Selection) (Fig. 1). The entire session lasted 20–30 min for adults.

## 3 Results

All data and the analysis scripts in R (version 4.2.2) are openly accessible (OSF: https://osf.io/yqd6g/). To test our main hypotheses, we first conducted generalized linear mixed effects models (GLMM) by using the R package *lme4* (v1.1‐26; Bates et al., 2015), compared models using likelihood ratio tests, and reported beta coefficients, standard errors, and *z*‐statistics.^3^ We then conducted a signal detection theory (SDT) analysis using the R package *basic*.

### 3.1 Main analysis

**Fig. 2:**
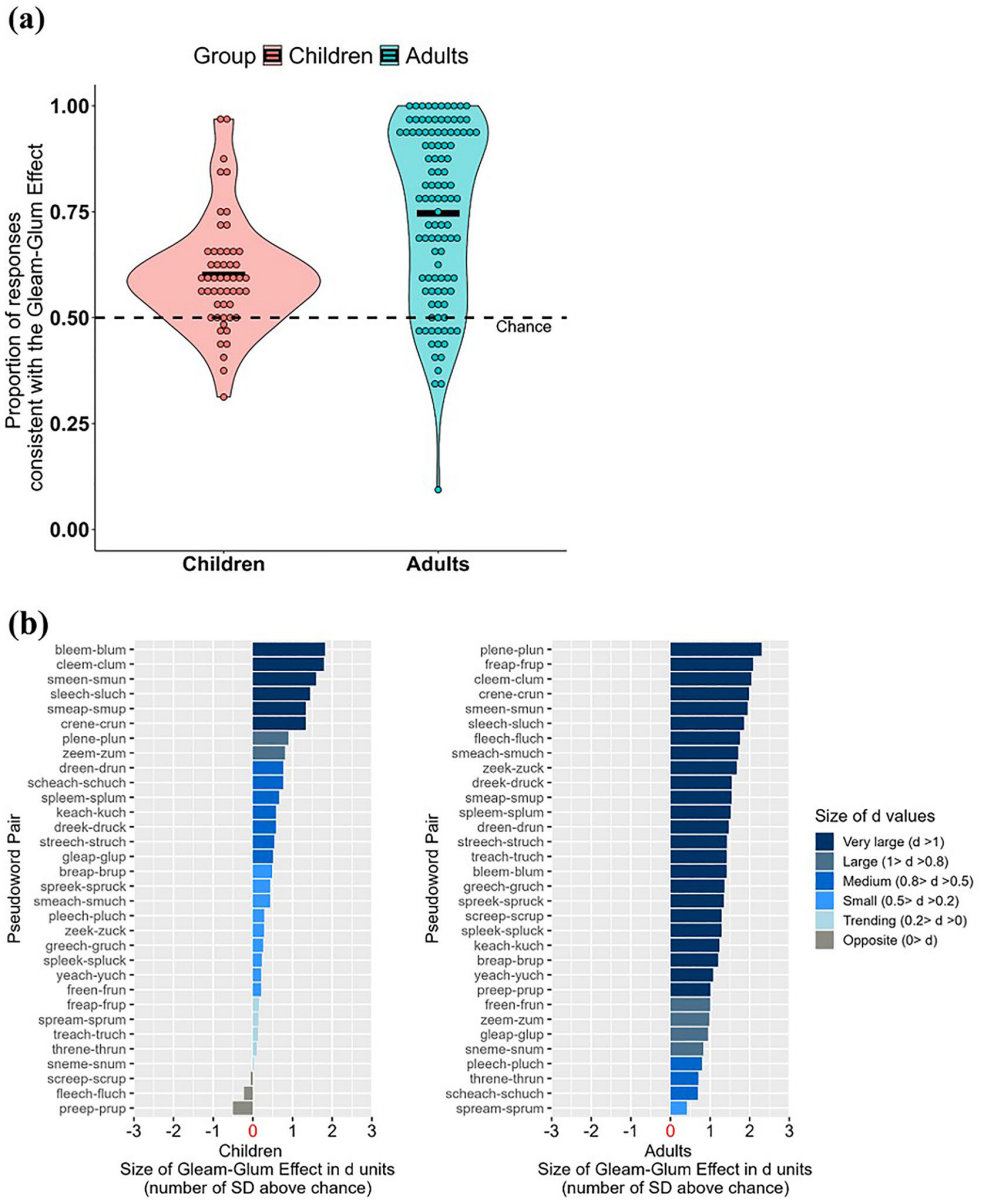
The gleam‐glum effect in the pseudoword‐to‐scene mapping task is plotted averaging (a) per participant in each age group and (b) per pseudoword pair in each age group. In (a), the x‐axis denotes age groups (children vs. adults). The y‐axis denotes the proportion of responses consistent with the gleam‐gleam effect for each participant, with 1 indicating the effect occurring across all 32 test trials (i.e., [i]‐pseudowords mapped with positive scenes and [Ʌ]‐pseudowords mapped with negative scenes 100% of the time), and 0 indicating the complete opposite of the effect across all 32 test trials. The dashed black line depicts the chance level (0.50) where [i]‐ and [Ʌ]‐pseudowords are each mapped with positive and negative scenes 50% of the time. The solid black bars denote the mean proportions by age group. Individual dots denote individual participants. In (b), the x‐axis shows the effect size of the gleam‐glum effect for each pseudoword pair averaged across participants, for children (left panel) and adults (right panel). The x‐axes denote the size of *d* values, indicating the number of standard deviations above chance in the direction consistent (or inconsistent) with the gleam‐glum effect. The y‐axes ordinally lists all 32 pseudoword pairs. The legend bars depict effect sizes at a pseudoword‐pair level.

Fig. 2a displays the proportions of responses consistent with the gleam‐glum effect for each participant (averaged across all test trials) for children and adults. We first assessed whether children matched the pseudowords to pictures of valence consistent with the gleam‐glum effect more often than chance (0.5). We conducted a GLMM model with trial‐by‐trial score as the outcome (1 represents responses that match [i]‐words with the positive drawing or [Ʌ]‐words with the negative drawing; 0 represents responses that do not match), and random intercepts for participant and item. The model specification was as follows: score for children ∼ 1 + logit(0.5) + (1 | Participant) + (1 | Item) (note that logit(0.5) is 0). Results showed that children's responses were significantly above chance and consistent with the gleam‐glum effect (*M* = 0.60, Δ*M_AboveChance_* = 0.10, *SD* = 0.13; *b* = 0.45, *STE* = 0.10, *z* = 4.37, *p* < .0001, 95% CI = [0.25, 0.66], odds ratio = 1.57). The random intercept for participant had a variance of 0.25 (*SD* = 0.50) and that for item had a variance of 0.20 (*SD* = 0.45), suggesting small variation across individuals and items (in typical behavioral research, variances of this magnitude—e.g., around 0.05–0.20—are often considered as small to moderate clustering effects, see Hox, Moerbeek, & van de Schoot, 2018; Snijders & Bosker, 2012). The strong effect provides the first evidence of the gleam‐glum effect among young children.

Next, we ran an exploratory analysis to examine if the magnitude of the gleam‐glum effect increased with age among the children. We generated one score per child by averaging across test trials and conducted a Pearson correlation with children's age. Results revealed that age did not significantly correlate with the gleam‐glum effect within this age range (*r* = 0.14, *p* = .34). In another exploratory analysis, we added age (mean‐centered) as a covariate to the GLMM model above. Children's performance remained significantly above chance level, and consistent with the correlation results, children's age was not a significant predictor of the gleam‐glum effect (see details in Supporting Information: https://osf.io/yqd6g/).

We also examined if adults showed the gleam‐glum effect more often than chance (0.5). Similar to children, we conducted the GLMM model with trial‐by‐trial score as the outcome (1, 0) and random intercepts for participant and item. The model specification was as follows: score for adults ∼ 1 + logit(0.5) + (1 | Participant) + (1 | Item). Results confirmed that adults’ responses were significantly above chance level and therefore consistent with the gleam‐glum effect (*M* = 0.75, *ΔM_AboveChance_* = 0.25, *SD* = 0.21; *b* = 1.51, *STE* = 0.15, *z* = 9.82, *p* < .0001, 95% CI = [1.21, 1.82], odds ratio = 4.53). The random intercept for participant had a variance of 1.92 (*SD* = 1.39) and that for item had a variance of 0.12 (*SD* = 0.34), suggesting substantial variation across individuals and relatively small item‐level variation. The strong effect among adults is consistent with prior work, which asked adult listeners to rate the valence of real words in English and Mandarin Chinese (Yu et al., 2021a, 2021b), and extends the gleam‐glum effect to carefully controlled and matched pseudowords that were linked with complex scenes depicting valence.

Next, we compared the magnitude of the gleam‐glum effect in children with that in adults. We conducted a GLMM model with trial‐by‐trial score as the outcome (1, 0), a fixed effect of age group (children vs. adults, contrast‐coded), random intercepts for participant and item, and a by‐item random slope for Age group. This was the final model specification: score for all participants ∼ Age group + (1 | Participant) + (1 + Age group | Item). Results revealed that adults displayed the gleam‐glum effect to a greater extent than children (*b* = 0.95, *STE* = 0.21, *z* = 4.48, *p* < .0001, 95% CI = [0.53, 1.38], odds ratio = 2.59). The random intercept for participant had a variance of 1.23 (*SD* = 1.11), indicating substantial variability in accuracy across individuals. The random intercept for items had a variance of 0.11 (*SD* = 0.33), suggesting small variation across items. The correlation between the random slope and intercept for items was 0.38, suggesting that items with higher accuracy tended to show a larger effect of Age group.^4^^,^ 5

### 3.2 Exploratory analyses

Additional analyses were conducted to assess the robustness of the gleam‐glum effect to account for scene selection biases. These analyses were not pre‐registered.

#### 3.2.1 Controlling for scene selection biases

We ran SDT analyses to separate any bias to favor selecting either positive or negative images from a pure measure of the gleam‐glum effect. SDT analyses provide two independent normalized metrics for each pseudoword pair, based on binomial probabilities. First, a bias metric, *c*, indicates how many standard deviations the group's selection of positive images for each pseudoword pair deviates from a chance level of 50%. Second, a separation metric, *d* (equivalent to Cohen's *d*), for each pseudoword pair, indicates how many standard deviations more the group favors positive images for [i]‐words than positive images for [Ʌ]‐words. The *c* value indicates the normalized bias to favor picking one type of emotional valence scene over the other, independent of whether the target word type is an [i]‐ or [Ʌ]‐pseudoword. Here, a negative *c* indicates a criterion shift to favor selecting more positive scenes, and a positive *c* indicates a shift to favor selecting more negative scenes. Because we calculated a *c* and *d* metric for each pseudoword pair, we then performed standard *t*‐test analyses on each of their distributions to provide an overall measure of positive versus negative image bias, along with an overall pure measure of the gleam‐glum effect independent of bias to favor selecting positive or negative scenes. This accounts for why *d* value effect sizes are typically larger than simple accuracy metrics, in particular when selection bias is present.

The SDT analyses for each pseudoword pair were conducted separately for children and adults. Results showed that children had a bias to match target words with positive scenes across the 32 pseudoword pairs (*c* = −0.16, *t*(31) = −3.48, *p* = .002, *d* = −0.62). Adults had a bias to match target words with negative scenes in general (*c* = 0.06, *t*(31) = 2.66, *p* = .012, *d* = 0.47). Importantly, the results confirmed that when this scene selection bias was controlled for, the gleam‐glum effect was large and remained significantly robust for both children (*M_d_* = 0.56, *SD* = 0.57, *t*(31) = 5.55, *p* < .0001, *d* = 0.98) and adults (*M_d_* = 1.37, *SD* = 0.46, *t*(31) = 17.02, *p* < .0001, *d* = 3.01). Further, the gleam‐glum effect size for adults (*M_d_* = 1.37, *SD* = 0.46) remained stronger than that for children (*M_d_* = 0.56, *SD* = 0.57) as revealed by an independent sample *t*‐test, *t*(62) = 6.24, *p* < .0001.

Fig. 2b displays the effect sizes (*d* values) for each pseudoword pair, indicating the number of standard deviations above chance in the direction consistent (or inconsistent) with the gleam‐glum effect for children (left panel) and adults (right panel). Across pseudoword pairs, the gleam‐glum effect was large and robust for children and adults. That is, for children, 75% of the pseudoword pairs individually demonstrated a small to very large gleam‐glum effect (with 90.6% in the predicted direction, and 25% exhibiting at least a large effect, *d* > 0.80). For adults, the effect applied to 100% of the pseudoword pairs tested in the current study, demonstrating a strong effect (with 100% in the predicted direction and 87.5% exhibiting at least a large effect, *d* > 0.80). Within each pseudoword pair, we can see commonalities and differences between age groups (Fig. 2b). Some pseudoword pairs consistently showed a large gleam‐glum effect for both children and adults (e.g., four of the same pseudoword pairs were among the top six pseudoword pairs on both lists, such as “*cleem*”–“*clum*”), whereas some other pseudoword pairs rendered quite different effect sizes across age groups (e.g., “*bleem*”–“*blum*” was ranked the first for children but the 16th for adults).

## 4 Discussion

The first aim was to develop a new methodology that allowed us to provide the first test of the gleam‐glum effect in both adults and young children. Results confirmed that both adults and 5‐ to 7‐year‐olds robustly exhibited the gleam‐glum effect: they reliably linked [i]‐pseudowords with scenes portraying positive valence and [Ʌ]‐pseudowords with scenes portraying negative valence, an effect of substantial magnitude. The second aim was to compare the gleam‐glum effect in children to that of adults. Results showed that although the gleam‐glum effect was strong in young children, it was even larger in adults. Together, these findings confirm for the first time that the gleam‐glum effect is robust in a sample of English‐speaking young children and college students and suggest that the effect strengthens with age. Further, the presence of the gleam‐glum effect at such a young age, when children are still building their vocabulary and honing their emotion processing skills, supports that this type of sound symbolic effect has promise to enhance language comprehension, language use, and language learning.

That young children systematically link pseudowords with the [i]‐phoneme to scenes conveying positive valence and pseudowords with the [Ʌ]‐phoneme to scenes conveying negative valence confirms for the first time the presence of emotional sound symbolism in young children. This finding transforms our understanding of sound symbolism in early childhood. Specifically, prior sound symbolism research largely consists of demonstrations of infants and children linking the sounds of words to physical properties of their meaning, such as size or shape (e.g., Fort et al., 2018; Imai & Kita, 2014; Maurer et al., 2006). Young children in the current study linked specific sounds to a more abstract feature of meaning—emotional valence. The gleam‐glum effect in young children demonstrates that at this stage of development, sound symbolism extends to a critical dimension for organizing meanings in the world and the words linked to those meanings (Vigliocco et al., 2009). Importantly, this association is not merely limited to a set of iconic words but extends to a large vocabulary of the English language, given the extensiveness of emotional expression, and the high frequency of usage of [i] and [Ʌ] vowel phonemes in English.

The presence of the gleam‐glum effect among 5‐ to 7‐year‐olds also highlights the strong link between language and emotion in early development (Hoemann, Xu, & Barrett, 2019). For example, in infancy, expressing language has been found to occur temporally close to expressing emotion (Bloom & Beckwith, 1989). Further, toddlers’ expressive language appears to support their development of emotion regulation (Cole, Armstrong, & Pemberton, 2010). Additionally, school‐aged children's receptive vocabulary has been linked to their knowledge and awareness of emotion (Beck, Kumschick, Eid, & Klann‐Delius, 2012). Language and emotional processing are still two domains that are undergoing development among 5‐ to 7‐year‐old children (Bloom, 2000; Declercq et al., 2019; Pons et al., 2004). That children in this age group demonstrate the gleam‐glum effect suggests that language and emotion could potentially work together to contribute to advancements in each domain.

In addition to providing the first evidence of the gleam‐glum effect in young children, results of the current study demonstrated, for the first time, differences in emotional sound symbolism between children and adults. Prior examinations of sound symbolism have largely only focused on young children or adults, rarely both (e.g., Kantartzis et al., 2011; Imai, Kita, Nagumo, & Okada, 2008; Tzeng et al., 2017), and we know less about how such biases change across development. One prior study on the bouba‐kiki sound symbolic effect among young children demonstrated that this effect increases as children age, with 3‐year‐olds showing a smaller effect, compared to 5‐ and 7‐year‐olds (Tzeng et al., 2017). The authors attributed this change to language experience. Our findings corroborate and expand on this prior notion, indicating that age‐related experience also plays a role in emotional sound symbolism: more experienced listeners (adults) demonstrated a stronger gleam‐glum effect than less experienced listeners (5‐ to 7‐year‐old children) in linking phonemes with emotional valence.

What kind of experience might strengthen the gleam‐glum effect? First, general experience with English words likely contributes to the strength of the gleam‐glum effect. Words across a large proportion of the English lexicon have been found to be consistent with the gleam‐glum effect, such that [i]‐phonemic words are generally rated with more positive valence, and [Ʌ]‐phonemic words with more negative valence (Yu et al., 2021a). So, the use of English over time may give rise to the gleam‐glum effect in 5‐ to 7‐year‐olds and strengthen this effect in college students. Second, more advanced cognitive skills that come with more experience are also a likely contributor. Children's cognitive skills are still developing, with adults having stronger memory, attention, and word learning abilities than children (e.g., Benitez & Li, 2024; Benitez & Robison, 2022; Vogan, Morgan, Powell, Smith, & Taylor, 2016). The task did place demands on memory and attention skills, as participants had to sustain their attention on the screen while hearing two presented words, and then selecting the image, out of two possible images, that could correspond to the test word. Thus, differences in memory and attention skills may have contributed to the differences between children and adults.

Furthermore, the strategies employed during the task may also change with more experience. In our method, both children and adults had to pick one out of two scenes of contrasting valence to match it with either an [i]‐ or [Ʌ]‐ pseudoword across multiple test trials. Adult listeners, who are more likely to exhibit explicit and rule‐based reasoning than children (Rabi, Miles, & Minda, 2015; Thomas et al., 2004), may have been more likely to map certain sounds with a category of pictures (e.g., [i]‐sounding pseudowords with all positive scenes), with less consideration of the mapping of individual sounds with individual scenes (see a similar argument in Monaghan, Mattock, & Walker, 2012). In contrast, child listeners may have been more likely to map individual sounds with individual scenes. Consistent with this conjecture are the findings that across items, adults showed less item‐level variation (such that on average, they responded significantly in the direction of the gleam‐glum effect for 100% of the pseudoword pairs, while children did so for only 75% of the pairs, see Fig. 2b). Further, across participants, adults showed more individual differences in task performance (i.e., compared to children, some adults were highly compliant with the gleam‐glum effect such that they reached 100% of responses consistent with the effect, while others were not, with only 15% of responses consistent with the effect, see Fig. 2a).

Another potential factor contributing to the age effect is that children exhibited a notable criterion bias to generally favor selecting positive pictures over negative ones. This type of bias can reduce overall accuracy. For example, if the positive picture was chosen on every trial, then every [i]‐word would produce results consistent with the gleam‐glum effect but none of the [Ʌ]‐words. This would result in 50% accuracy and no overall gleam‐glum effect. The results with children showed a robust gleam‐glum effect, but this effect could have been dampened by the positive scene bias if on some trials, children's tendency to favor positive pictures led them to select the positive picture without consideration of the individual words presented.

Finally, while children showed a bias to favor picking positive images more often than chance, adults favored the negative images more often than chance. This brings to light likely emotional differences in reasoning the change developmentally between these two age groups. For example, children may be accustomed to encountering a higher degree of positive imagery and generally have a positive reaction to hearing funny‐sounding novel words, whereas adults may be more wary and tend to generally find novel words somewhat distressing. Critically, our results that indicate differences between children and adults in scene selection biases can inform our understanding of the underlying mechanisms of emotional sound symbolism. Altogether, findings point to that a comprehensive theoretical model of emotional sound symbolism must account for the role of age‐related experience.

What are the mechanisms that underlie the gleam‐glum effect? There are several possibilities. One mechanism is that exhibiting emotions (i.e., smiling) and pronouncing certain phonemes (i.e., pronouncing [i]) share the same orofacial musculature (e.g., Yu et al., 2021a; Körner & Rummer, 2022; Rummer et al., 2014; Rummer & Schweppe, 2019). Specifically, the typical motor actions in pronouncing [i] involves contracting the zygomaticus muscle used to smile, while those used to produce [Ʌ] inhibit that muscle and may contract other muscles (e.g., suprahyoid muscles under the chin; Wang & Yiu, 2023), and may require tongue lowering and retraction when producing central vowels ([Ʌ] as a central vowel) with the involvement of tongue muscles like the genioglussus (e.g., Takano & Honda, 2007). Thus, emotion and language systems may be grounded by a common underlying orofacial musculature, such that emotional state and language production or perception are linked bi‐directionally via orofacial articulation (e.g., Havas, Glenberg, & Rinck, 2007; Hirayama, 2016; Imai et al., 2025; Rummer et al., 2014; Sidequersky et al., 2016). These ideas are in line with a recent proposal, the articulatory feedback hypothesis, suggesting that emotion and vowel phonemes are similarly connected via orofacial muscle movements, which then provide articulatory feedback to the speakers on their emotional state (see Körner & Rummer, 2022, 2023; Rummer et al., 2014). Evidence in favor of this proposal has demonstrated that adults interpreted cartoons as funnier when either moving the zygomaticus major muscle involved in smiling (e.g., holding a pen with the teeth) or producing vowels that require such muscle movements (e.g., uttering [i]), compared to when engaging in an action (e.g., holding a pen with the lips) or producing other vowels (e.g., uttering [o] in German) that inhibit the movements of zygomaticus major muscle (see Rummer et al., 2014; Rummer & Schweppe, 2019; Yu et al., 2021a).^6^ Furthermore, restricting the orofacial movements of adults with hearing loss affects their judgement on sound‐shape mappings, supporting the linkage between sound symbolism and articulation (Imai et al., 2025). Therefore, the overlap between producing language and expressing emotions may strengthen the link between phonemes and the valence of words containing those phonemes.

Another potential explanation underlying the gleam‐glum effect is that acoustic differences across vowels may be linked to emotion (e.g., Rummer et al., 2022). For instance, [i]‐phonemes are naturally produced with a higher pitch than [Ʌ]‐phonemes (Patten & McBeath, 2025). Higher pitch, especially in women's speech, is rated as more attractive and as more positive in valence by adults (e.g., Niculescu, Van Dijk, Nijholt, Li, & See, 2013; Re, O'Connor, Bennett, & Feinberg, 2012), is preferred by preverbal infants (e.g., Masapollo, Polka, & Ménard, 2016), and has been found to be prevalent in infant‐directed speech (e.g., Narayan & McDermott, 2016). In an exploratory analysis, we assessed if there were systematic pitch differences between the pseudoword pairs used in our task. We found that the paired [i]‐ and [Ʌ]‐pseudowords were comparable in pitch (see Footnote 2). Thus, the current study suggests that acoustic properties of pitch *alone* do not seem to be notable or necessary drivers of the gleam‐glum effect. Furthermore, the finding that there can be systematic pitch differences between phonemes could be also viewed as an epiphenomenon of other underlying factors such as a product of the different orofacial musculature used to produce the phonemes. Thus, pitch differences, if any, could be interpreted as a parallel physical manifestation, complementary to the articulatory production rather than as an alternative explanation of sound symbolism. In any case, findings from the current study pave the way for further exploration of the mechanisms that promote the gleam‐glum effect across development.

The robustness of the gleam‐glum effect demonstrated in the current study also encourages future work to assess whether the effect is driven by both phonemes tested or is largely driven by one of the phonemes. It is possible that the phoneme‐valence link is more prevalent for one phoneme type (e.g., [i]) than the other (e.g., [Ʌ]). For instance, the broader sound symbolic literature suggests an asymmetric link in the bouba‐kiki effect, such that the link between “*bouba*” and a round shape is more robust than that between “*kiki*” and a spiky shape in early infancy (e.g., Fort et al., 2018). More related to the current work, prior studies assessing the valence contrast between the [i]‐[o] phonemes in German suggest that the [i]‐phoneme‐to‐positive‐valence link is more robust than the [o]‐phoneme‐to‐negative‐valence link (Körner & Rummer, 2023). Given that pronouncing both [o] and [Ʌ] render some similar and some differing orofacial muscular movements (e.g., both inhibit the zygomaticus major muscle contractions but otherwise differ; see Rummer et al., 2014; Yu et al., 2021a), one possibility is that the [i]‐phoneme‐to‐positive‐valence is the primary contributor to the gleam‐glum effect. Another possibility is that both vowels may equally contribute to the gleam‐glum effect or even that [Ʌ] could be the major contributor in the English language (Adelman et al., 2018; Householder, 1946; Taylor & Taylor, 1965). Evidence consistent with the latter conjecture includes ratings of the valences of the lexicon of English words (see Warriner et al., 2013; Yu et al., 2021a), which reveals that on average, real words that contain the [Ʌ]‐phoneme are rated as slightly more negative by English‐speaking adults (*M* = 4.83, ranging from 1 the most negative to 9 the most positive, with the mean of 5), compared to those containing the [i]‐phoneme (*M* = 5.12). The aggregate of findings suggests that both [i]‐ and [Ʌ]‐phonemes have promise to contribute to the gleam‐glum effect.

Our findings are the first to provide empirical evidence that children as young as 5‐ to 7‐year‐olds exhibit a robust gleam‐glum effect. These findings serve as the cornerstone for future work to assess if the effect emerges even earlier in childhood. The bouba‐kiki effect has been found across adults (Ramachandra & Hubbard, 2001; Ćwiek et al., 2022), toddlers (Imai et al., 2015; Maurer et al., 2006), and preverbal infants as young as 4‐month‐olds (e.g., Ozturk, Krehm, & Vouloumanos, 2013), though the effect is not always demonstrated at younger ages (see Pejovic & Molnar, 2017; Sidhu et al., 2023). A key prerequisite to the emotional sound symbolic effect is the sensitivity to interpret emotion in the real world. Infants can detect emotional expressions in their parents by 6 months of age (Walker‐Andrews, 1998) and can associate emotions with actions by 9 months of age (Barna & Legerstee, 2005). Indeed, the speech directed at infants and children in early development often conveys accentuated emotion (Fernald, 1985; Trainor, Austin, & Desjardins, 2000). Further, in the first year of life, infants readily express emotion such as smiling and crying via facial‐muscular movements (Illingworth, 1955; Messinger, 2002) and produce a variety of phonemes including [i]‐vowels (Buhr, 1980; Lieberman, 1980), which suggests that the cross‐modal links among facial musculature, emotions, and phonemes may be formed in infancy (e.g., Li & Benitez, 2025). In this vein, it is sensible to speculate that the gleam‐glum effect emerges earlier than tested here. Our study provides a solid methodological foundation and empirical support to test whether the language‐emotion link is present in even younger children.

The presence of the gleam‐glum effect in young children suggests that the effect has promise to promote early language learning. Prior work demonstrates that early sensitivity to other sound symbolic patterns may scaffold word learning (e.g., Imai & Kita, 2014), though the scope of such a bootstrapping effect remains under debate—that is, whether it supports learning of iconic words narrowly or words in general including other less iconic words (Nielsen & Dingemanse, 2021). Our work demonstrates a strong bias in young children and adults to map [i]‐phonemic words with positive valence and [Ʌ]‐phonemic words with negative valence. This type of sound–emotion association, similar to the other sound symbolic patterns, may serve as a fundamental bootstrapping mechanism for young children to learn novel words. That is, sensitivity to the sound–emotion associations may boost learners to acquire new words containing these specific phonemic features (i.e., a wide set of words in English contains vowels involved in the *gleam‐glum* effect) or leverage learners’ overall vocabulary not limited to words of these features (i.e., established early word‐referent mappings reduce referential ambiguity and narrow down the scope of meanings of other new words, see Hidaka & Smith, 2010). Our findings lay a foundation for future work to empirically test whether the gleam‐glum effect facilitates real‐time word learning across development—for example if mappings between words with the [i]‐phoneme and positive valence, and those with the [Ʌ]‐phoneme and negative valence, are easier to learn than mappings inconsistent with the effect (see an example in Monaghan et al., 2012).

## 5 Conclusion

In summary, our study introduces a novel pseudoword‐to‐scene matching methodology that supports the robustness of an emerging type of sound symbolism—the gleam‐glum effect—where phonemes systematically carry emotional valence. Both English‐speaking 5‐ to 7‐year‐old children and adults robustly matched pseudowords containing the [i]‐phoneme to emotionally positive scenes, and pseudowords containing the [Ʌ]‐phoneme to emotionally negative scenes. The robustness of the gleam‐glum effect in young children and its generalizability spanning many words expand our understanding of sound symbolism. The results indicate that it extends beyond physical properties to more abstract aspects of meaning, and that language and emotion are interconnected in early development. Further, adults displayed a stronger bias than children, implicating age‐related experience as one important mechanism underlying the gleam‐glum effect. Overall, our findings demonstrate that young children and adults have a robust bias to link phonemes with valence, suggesting that emotional sound symbolism has the promise of being an important mechanism for language comprehension, language use, and language learning.

## Supporting information



Supporting Information

## Data Availability

This research received approval from the IRB committee at Arizona State University (IDs: STUDY00007151 Language Learning in Adults, and STUDY00015502 Sound Symbolism in Children). The hypotheses and methods were pre‐registered (for adults: https://doi.org/10.17605/OSF.IO/AZQXU on 2022‐05‐12; for children: https://doi.org/10.17605/OSF.IO/TU7J3 on 2022‐04‐08), both prior to data collection. The data analysis plan was pre‐registered but changed during the manuscript writing (see reasons in the manuscript, Footnote 3). The results presented in the manuscript were consistent with the results from the preregistration (see the Supporting Information, https://osf.io/yqd6g/). Upon publication, the data, analysis scripts, and study materials will be shared with the public, including supplementary, raw data, the R analysis scripts, and the PsychoPy program used to run the experiments (https://osf.io/yqd6g/).

## Notes

1 Other earlier research in emotional sound symbolism has with some success explored the distinction between [i] versus [o] phonemes consistent with an articulatory feedback hypothesis (Körner & Rummer, 2022, 2023; Rummer, Schweppe, Schlegelmilch, & Grice, 2014), but for several reasons here, we elected to pursue the gleam‐glum [i] versus [Ʌ] phoneme distinction, including that it appears promising to produce a very robust effect in the tested English language.

2 We obtained the mean pitch information from each pseudoword using Praat software (https://www.fon.hum.uva.nl/praat/) and compared the pitches of all [i]‐pseudowords with those of all [Ʌ]‐pseudowords via a paired sample *t*‐test. Results showed that [i]‐pseudowords (*M* = 272.28 Hz, *SD* = 44.56) and [Ʌ]‐pseudowords (*M* = 261.01 Hz, *SD* = 38.86) did not differ significantly in pitch, *t*(62) = 1.08, *p* = .285. Then, we assessed the pitch differences within each pseudoword pair by subtracting the pitch of the [i]‐pseudoword (e.g., *zeem*) by the pitch of the [Ʌ]‐pseudoword (e.g., *zum*). Within each pseudoword pair, a positive value denoted a numerically higher pitch of the [i]‐pseudoword, and a negative value denoted a numerically higher pitch of the [Ʌ]‐pseudoword; zero denoted no differences in pitch. Out of the 32 pseudoword pairs, 18 pairs had the [i]‐pseudoword higher in pitch, with the others having the [Ʌ]‐pseudoword higher in pitch. Via a one‐sample *t*‐test comparing these pitch differences against zero, results showed that the pitch differences within pseudoword pairs did not significantly differ from zero (*t*(31) = 1.29, *p* = .207), suggesting that [i]‐ and [Ʌ]‐pseudowords were similar in overall pitch.

3 Our pre‐registered analysis plan was to conduct *t*‐tests and ANOVA tests (see pre‐registration in OSF, for children: https://doi.org/10.17605/OSF.IO/TU7J3; and for adults https://doi.org/10.17605/OSF.IO/AZQXU). We opted to report our final data using generalized linear mixed effect models, as this more conservative approach accounts for the binary accuracy outcome (1, 0), for random effects of items, and variance across individuals. The results of the pre‐registered plan (see Supporting Information in OSF: https://osf.io/yqd6g/) are consistent with the results reported in the manuscript.

4 We additionally explored whether the alignment between the visually presented images (left or right images) and the aurally presented words (first or second words) impacted the task performance across all participants as suggested by a reviewer. Results showed the gleam‐glum effect was still robust when accounting for word and picture order alignment. See Supporting Information (https://osf.io/yqd6g/)

5 In an exploratory analysis, we assessed whether the gleam‐glum effect differed by vowel type ([i]‐ versus [Ʌ]‐ pseudowords) as suggested by reviewers. Results showed that the effect was comparable in both vowels (see details in the Supporting Information, https://osf.io/yqd6g/). Yet, this analysis shall be interpreted with caution due to the reasons mentioned in the Supporting Information.

6 Much of the original work primarily tested the [o] phoneme as the negative example used to compare against the positive [i] one. Additionally, there is evidence that the [o] phoneme is associated with contracting the depressor anguli oris muscle used in one type of negative emotion: frowning. Here, we note that there are multiple generally recognizable negative emotions, each with their unique orofacial musculature, and that our current and previous gleam‐glum effect findings are consistent with including the [Ʌ] phoneme as one of them.

## References

[cogs70215-bib-0001] Adelman, J. S. , Estes, Z. , & Cossu, M. (2018). Emotional sound symbolism: Languages rapidly signal valence via phonemes. Cognition, 175, 122–130. 10.1016/j.cognition.2018.02.007 29510337

[cogs70215-bib-0002] Aryani, A. , Conrad, M. , Schmidtke, D. , & Jacobs, A. (2018). Why ‘piss’ is ruder than ‘pee’? The role of sound in affective meaning making. PloS One, 13(6), e0198430. 10.1371/journal.pone.0198430 29874293 PMC5991420

[cogs70215-bib-0003] Asano, M. , Imai, M. , Kita, S. , Kitajo, K. , Okada, H. , & Thierry, G. (2015). Sound symbolism scaffolds language development in preverbal infants. Cortex, 63, 196–205. 10.1016/j.cortex.2014.08.025 25282057

[cogs70215-bib-0004] Baldwin, D. A. (1993). Early referential understanding: Infants’ ability to recognize referential acts for what they are. Developmental Psychology, 29(5), 832–843. 10.1037/0012-1649.29.5.832

[cogs70215-bib-0005] Barber, H. , & Reimer, T. (2021). The influence of speaker pitch on inferring semantic valence. Metaphor and Symbol, 36(2), 63–73. 10.1080/10926488.2021.1875322

[cogs70215-bib-0006] Barna, J. , & Legerstee, M. (2005). Nine‐and twelve‐month‐old infants relate emotions to people's actions. Cognition & Emotion, 19(1), 53–67. https://doi‐org.ezproxy1.lib.asu.edu/10.1080/02699930341000021

[cogs70215-bib-0007] Bates, D. , Mächler, M. , Bolker, B. , & Walker, S. (2015). Fitting Linear Mixed‐Effects Models Using lme4. Journal of Statistical Software, 67(1), 1–48. 10.18637/jss.v067.i01

[cogs70215-bib-0008] Beck, L. , Kumschick, I. R. , Eid, M. , & Klann‐Delius, G. (2012). Relationship between language competence and emotional competence in middle childhood. Emotion, 12(3), 503–514. 10.1037/a0026320 22148995

[cogs70215-bib-0009] Benitez, V. L. , & Li, Y. (2024). Cross‐situational word learning in children and adults: The case of lexical overlap. Language Learning and Development, 20(3), 195–218. 10.1080/15475441.2023.2256713

[cogs70215-bib-0010] Benitez, V. L. , & Robison, M. K. (2022). Pupillometry as a window into young children's sustained attention. Journal of Intelligence, 10(4), 107. 10.3390/jintelligence10040107 36412787 PMC9680391

[cogs70215-bib-0011] Benitez, V. L. , & Saffran, J. R. (2018). Predictable events enhance word learning in toddlers. Current Biology, 28(17), 2787–2793. 10.1016/j.cub.2018.06.017 30122525 PMC6148368

[cogs70215-bib-0012] Blasi, D. E. , Wichmann, S. , Hammarström, H. , Stadler, P. F. , & Christiansen, M. H. (2016). Sound‐meaning association biases evidenced across thousands of languages. Proceedings of the National Academy of Sciences, 113(39), 10818–10823. 10.1073/pnas.1605782113 PMC5047153 27621455

[cogs70215-bib-0013] Bloom, P. (2000). How children learn the meanings of words. Cambridge: MIT Press. 10.1017/s0140525x01000139 12412326

[cogs70215-bib-0014] Bloom, L. , & Beckwith, R. (1989). Talking with feeling: Integrating affective and linguistic expression in early language development. Cognition & Emotion, 3(4), 313–342. 10.1080/02699938908412711

[cogs70215-bib-0905] Bridges, D. , Pitiot, A. , MacAskill, M. R. , & Peirce, J. W. (2020). The timing mega‐study: Comparing a range of experiment generators, both lab‐based and online. PeerJ, 8, e9414. 10.7717/peerj.9414 33005482 PMC7512138

[cogs70215-bib-0016] Buhr, R. D. (1980). The emergence of vowels in an infant. Journal of Speech, Language, and Hearing Research, 23(1), 73–94. 10.1044/jshr.2301.73 7442186

[cogs70215-bib-0017] Cole, P. M. , Armstrong, L. M. , & Pemberton, C. K. (2010). The role of language in the development of emotion regulation. In S. D. Calkins & M. A. Bell (Eds.), Child development at the intersection of emotion and cognition (pp. 59–77). Washington, DC: American Psychological Association. 10.1037/12059-004

[cogs70215-bib-0018] Conrad, M. , Ullrich, S. , Schmidtke, D. , & Kotz, S. A. (2022). ERPs reveal an iconic relation between sublexical phonology and affective meaning. Cognition, 226, 105182. 10.1016/j.cognition.2022.105182 35689874

[cogs70215-bib-0019] Ćwiek, A. , Fuchs, S. , Draxler, C. , Asu, E. L. , Dediu, D. , Hiovain, K. , Kawahara, S. , Koutalidis, S. , Krifka, M. , Lippus, P. , Lupyan, G. , Oh, G. E. , Paul, J. , Petrone, C. , Ridouane, R. , Reiter, S. , Schümchen, N. , Szalontai, A. , Ünal‐Logacev, O. , Zeller, J. , Perlman, M. , & Winter, B. (2022). The *bouba/kiki* effect is robust across cultures and writing systems. Philosophical Transactions of the Royal Society B: Biological Sciences, 377(1841), 20200390. 10.1098/rstb.2020.0390 PMC8591387 34775818

[cogs70215-bib-0020] Darwin, C. R. (1872). The expression of the emotions in man and animals. London: John Murray.

[cogs70215-bib-0021] de Saussure, F. (1916). Course in general linguistics. London: McGraw‐Hill.

[cogs70215-bib-0022] Declercq, C. , Marlé, P. , & Pochon, R. (2019). Emotion word comprehension in children aged 4–7 years. The Educational and Developmental Psychologist, 36(2), 82–87. 10.1017/edp.2019.17

[cogs70215-bib-0024] Fernald, A. (1985). Four‐month old infants prefer to listen to motherese. Infant Behavior and Development, 8, 181–195. 10.1016/S0163-6383(85)80005-9

[cogs70215-bib-0025] Fisher, C. , Gertner, Y. , Scott, R. M. , & Yuan, S. (2010). Syntactic bootstrapping. WIREs Cognitive Science, 1(2), 143–149. 10.1002/wcs.17 26271229

[cogs70215-bib-0026] Filippi, P. (2020). Emotional voice intonation: A communication code at the origins of speech processing and word‐meaning associations. Journal of Nonverbal Behavior, 44(4), 395–417. 10.1007/s10919-020-00337-z

[cogs70215-bib-0027] Fort, M. , Lammertink, I. , Peperkamp, S. , Guevara‐Rukoz, A. , Fikkert, P. , & Tsuji, S. (2018). Symbouki: A meta‐analysis on the emergence of sound symbolism in early language acquisition. Developmental Science, 21(5), e12659. 10.1111/desc.12659 29542266

[cogs70215-bib-0029] Garrido, M. V. , & Godinho, S. (2021). When vowels make us smile: The influence of articulatory feedback in judgments of warmth and competence. Cognition and Emotion, 35(5), 837–843. 10.1080/02699931.2021.1900076 33745414

[cogs70215-bib-0031] Gleitman, L. (1990). The structural sources of verb meanings. Language Acquisition, 1(1), 3–55. https://doi‐org.ezproxy1.lib.asu.edu/10.1207/s15327817la0101_2

[cogs70215-bib-0032] Havas, D. A. , Glenberg, A. M. , & Rinck, M. (2007). Emotion simulation during language comprehension. Psychonomic Bulletin & Review, 14(3), 436–441. 10.3758/BF03194085 17874584

[cogs70215-bib-0033] Hidaka, S. , & Smith, L. B. (2010). A single word in a population of words. Language Learning and Development, 6(3), 206–222. 10.1080/15475441.2010.484380 26097439 PMC4469392

[cogs70215-bib-0034] Hirayama, M. J. (2016). A physical figure model of lips for speech production. The Journal of the Acoustical Society of America, 140(4), 3004–3004. 10.1121/1.4969314

[cogs70215-bib-0035] Hoemann, K. , Xu, F. , & Barrett, L. F. (2019). Emotion words, emotion concepts, and emotional development in children: A constructionist hypothesis. Developmental Psychology, 55(9), 1830–1849. 10.1037/dev0000686 31464489 PMC6716622

[cogs70215-bib-0036] Householder, F. W., Jr. (1946). On the problem of sound and meaning, and English phonesthemes. Word, 2(2), 83–84. 10.1080/00437956.1946.11659295

[cogs70215-bib-0037] Hox, J. J. , Moerbeek, M. , & van de Schoot, R. (2018). Multilevel analysis: Techniques and applications (3rd ed.). New York: Routledge.

[cogs70215-bib-0038] Illingworth, R. S. (1955). Crying in infants and children. British Medical Journal, 1(4905), 75–78. 10.1136/bmj.1.4905.75 13219346 PMC2060770

[cogs70215-bib-0039] Imai, M. , & Kita, S. (2014). The sound symbolism bootstrapping hypothesis for language acquisition and language evolution. Philosophical Transactions of the Royal Society B: Biological Sciences, 369(1651), 20130298. 10.1098/rstb.2013.0298 PMC4123677 25092666

[cogs70215-bib-0907] Imai, M. , Kita, S. , Akita, K. , Saji, N. , Ohba, M. , & Namatame, M. (2025). Does sound symbolism need sound? The role of articulatory movement in detecting iconicity between sound and meaning. The Journal of the Acoustical Society of America, 157(1), 137–148. 10.1121/10.0034832 39791996

[cogs70215-bib-0040] Imai, M. , Kita, S. , Nagumo, M. , & Okada, H. (2008). Sound symbolism facilitates early verb learning. Cognition, 109(1), 54–65. 10.1016/j.cognition.2008.07.015 18835600

[cogs70215-bib-0041] Imai, M. , Miyazaki, M. , Yeung, H. H. , Hidaka, S. , Kantartzis, K. , Okada, H. , & Kita, S. (2015). Sound symbolism facilitates word learning in 14‐month‐olds. PloS One, 10(2), e0116494. 10.1371/journal.pone.0116494 25695741 PMC4335030

[cogs70215-bib-0042] Jablonka, E. , Ginsburg, S. , & Dor, D. (2012). The co‐evolution of language and emotions. Philosophical Transactions of the Royal Society B: Biological Sciences, 367(1599), 2152–2159. 10.1098/rstb.2012.0117 PMC3385682 22734058

[cogs70215-bib-0043] Jo, J. , & Ko, E. S. (2018). Korean mothers attune the frequency and acoustic saliency of sound symbolic words to the linguistic maturity of their children. Frontiers in Psychology, 9, 2225. 10.3389/fpsyg.2018.02225 30618893 PMC6305434

[cogs70215-bib-0044] Johansson, N. , & Zlatev, J. (2013). Motivations for sound symbolism in spatial deixis: A typological study of 101 languages. Public Journal of Semiotics, 5(1), 3–20. 10.37693/pjos.2013.5.9668

[cogs70215-bib-0045] Kambara, T. , & Umemura, T. (2021). The relationships between initial consonants in Japanese sound symbolic words and familiarity, multi‐sensory imageability, emotional valence, and arousal. Journal of Psycholinguistic Research, 50(4), 831–842. 10.1007/s10936-020-09749-w 33394300

[cogs70215-bib-0046] Kamiloğlu, R. G. , Fischer, A. H. , & Sauter, D. A. (2020). Good vibrations: A review of vocal expressions of positive emotions. Psychonomic Bulletin & Review, 27, 237–265. 10.3758/s13423-019-01701-x 31898261 PMC7093353

[cogs70215-bib-0047] Kantartzis, K. , Imai, M. , & Kita, S. (2011). Japanese sound‐symbolism facilitates word learning in English‐speaking children. Cognitive Science, 35(3), 575–586. 10.1111/j.1551-6709.2010.01169.x

[cogs70215-bib-0048] Körner, A. , & Rummer, R. (2022). Articulation contributes to valence sound symbolism. Journal of Experimental Psychology: General, 151(5), 1107–1114. 10.1037/xge0001124 34694857

[cogs70215-bib-0049] Körner, A. , & Rummer, R. (2023). Valence sound symbolism across language families: A comparison between native speakers of Japanese and German. Language and Cognition, 15(2), 337–354. 10.1017/langcog.2022.39

[cogs70215-bib-0050] Laing, C. (2019). A role for onomatopoeia in early language: Evidence from phonological development. Language and Cognition, 11(2), 173–187. 10.1017/langcog.2018.23

[cogs70215-bib-0051] Li, Y. , & Benitez, V. L. (2025). Concurrences across time and sensorimotor capacities promote infant learning. Child Development Perspectives, 19(2), 99–107. 10.1111/cdep.12531

[cogs70215-bib-0052] Lieberman, P. (1980). On the development of vowel productions in young children. In G. Yeni‐Komshian , J. F. Kavanagh , & C. A. Ferguson (Eds.), Child phonology, Vol. 1: Production (pp. 113–142). New York: Academic Press.

[cogs70215-bib-0053] Lockwood, G. , & Dingemanse, M. (2015). Iconicity in the lab: A review of behavioral, developmental, and neuroimaging research into sound‐symbolism. Frontiers in Psychology, 6, 1246. 10.3389/fpsyg.2015.01246 26379581 PMC4547014

[cogs70215-bib-0054] Lupyan, G. , & Casasanto, D. (2015). Meaningless words promote meaningful categorization. Language and Cognition, 7(2), 167–193. 10.1017/langcog.2014.21

[cogs70215-bib-0055] Majid, A. (2012). Current emotion research in the language sciences. Emotion Review, 4(4), 432–443. 10.1177/1754073912445827

[cogs70215-bib-0056] Masapollo, M. , Polka, L. , & Ménard, L. (2016). When infants talk, infants listen: Pre‐babbling infants prefer listening to speech with infant vocal properties. Developmental Science, 19(2), 318–328. 10.1111/desc.12298 25754812

[cogs70215-bib-0057] Maurer, D. , Pathman, T. , & Mondloch, C. J. (2006). The shape of boubas: Sound–shape correspondences in toddlers and adults. Developmental Science, 9(3), 316–322. 10.1111/j.1467-7687.2006.00495.x 16669803

[cogs70215-bib-0058] Messinger, D. S. (2002). Positive and negative: Infant facial expressions and emotions. Current Directions in Psychological Science, 11(1), 1–6. 10.1111/1467-8721.00156

[cogs70215-bib-0059] Monaghan, P. , Christiansen, M. H. , & Fitneva, S. A. (2011). The arbitrariness of the sign: Learning advantages from the structure of the vocabulary. Journal of Experimental Psychology: General, 140(3), 325–347. 10.1037/a0022924 21517205

[cogs70215-bib-0060] Monaghan, P. , Mattock, K. , & Walker, P. (2012). The role of sound symbolism in language learning. Journal of Experimental Psychology: Learning, Memory, and Cognition, 38(5), 1152. 10.1037/a0027747 22468804

[cogs70215-bib-0061] Monaghan, P. , Shillcock, R. C. , Christiansen, M. H. , & Kirby, S. (2014). How arbitrary is language? Philosophical Transactions of the Royal Society B: Biological Sciences, 369(1651), 20130299. 10.1098/rstb.2013.0299 PMC4123678 25092667

[cogs70215-bib-0062] Narayan, C. R. , & McDermott, L. C. (2016). Speech rate and pitch characteristics of infant‐directed speech: Longitudinal and cross‐linguistic observations. The Journal of the Acoustical Society of America, 139(3), 1272–1281. 10.1121/1.4944634 27036263

[cogs70215-bib-0063] Nencheva, M. L. , Tamir, D. I. , & Lew‐Williams, C. (2023). Caregiver speech predicts the emergence of children's emotion vocabulary. Child Development, 94(3), 585–602. 36852506 10.1111/cdev.13897 PMC10121903

[cogs70215-bib-0064] Niculescu, A. , Van Dijk, B. , Nijholt, A. , Li, H. , & See, S. L. (2013). Making social robots more attractive: The effects of voice pitch, humor and empathy. International Journal of Social Robotics, 5, 171–191. 10.1007/s12369-012-0171-x

[cogs70215-bib-0901] Nielsen, A. K. , & Dingemanse, M. (2021). Iconicity in word learning and beyond: A critical review. Language and Speech, 64(1), 52–72. 10.7717/0023830920914339 32308121 PMC7961653

[cogs70215-bib-0065] Ozturk, O. , Krehm, M. , & Vouloumanos, A. (2013). Sound symbolism in infancy: Evidence for sound–shape cross‐modal correspondences in 4‐month‐olds. Journal of Experimental Child Psychology, 114(2), 173–186. 10.1016/j.jecp.2012.05.004 22960203

[cogs70215-bib-0066] Patten, K. J. , McBeath, M. K. , & Baxter, L. C. (2019). Harmonicity: Behavioral and neural evidence for functionality in auditory scene analysis, Auditory Perception & Cognition, 1(3‐4), 150–172. 10.1080/25742442.2019.1609307

[cogs70215-bib-0067] Patten, K. J. , & McBeath, M. K. (2020). The difference between shrieks and shrugs: Spectral envelope correlates with changes in pitch and loudness. Proceedings of the 2nd International Conference on Timbre (Timbre 2020) . Thessaloniki, Greece.

[cogs70215-bib-0068] Patten, K. J. , & McBeath, M. K. (2025). Scat singing and vocal winging: Vowel phoneme timbre dimensions reflect physical natural regularities. Auditory Perception & Cognition, 7(4), 1–33. 10.1080/25742442.2024.2435235

[cogs70215-bib-0069] Paul, E. S. , Sher, S. , Tamietto, M. , Winkielman, P. , & Mendl, M. T. (2020). Towards a comparative science of emotion: Affect and consciousness in humans and animals. Neuroscience & Biobehavioral Reviews, 108, 749–770. 10.1016/j.neubiorev.2019.11.014 31778680 PMC6966324

[cogs70215-bib-0904] Peirce, J. W. , Gray, J. R. , Simpson, S. , MacAskill, M. R. , Höchenberger, R. , Sogo, H. , Kastman, E. , & Lindeløv, J. K. (2019). PsychoPy2: Experiments in behavior made easy. Behavior Research Methods, 51(1), 195–203. 10.3758/s13428-018-01193-y 30734206 PMC6420413

[cogs70215-bib-0070] Pejovic, J. , & Molnar, M. (2017). The development of spontaneous sound–shape matching in monolingual and bilingual infants during the first year. Developmental Psychology, 53(3), 581–586. 10.1037/dev0000274 27854461

[cogs70215-bib-0071] Perry, L. K. , Perlman, M. , & Lupyan, G. (2015). Iconicity in English and Spanish and its relation to lexical category and age of acquisition. PloS One, 10(9), e0137147. 10.1371/journal.pone.0137147 26340349 PMC4560417

[cogs70215-bib-0072] Perry, L. K. , Custode, S. A. , Fasano, R. M. , Gonzalez, B. M. , & Savy, J. D. (2021). What is the buzz about iconicity? How iconicity in caregiver speech supports children's word learning. Cognitive Science, 45(4), e12976. 10.1111/cogs.12976 33873243

[cogs70215-bib-0073] Pons, F. , Harris, P. L. , & De Rosnay, M. (2004). Emotion comprehension between 3 and 11 years: Developmental periods and hierarchical organization. European Journal of Developmental Psychology, 1(2), 127–152. 10.1080/17405620344000022

[cogs70215-bib-0074] Rabaglia, C. D. , Maglio, S. J. , Krehm, M. , Seok, J. H. , & Trope, Y. (2016). The sound of distance. Cognition, 152, 141–149. 10.1016/j.cognition.2016.04.001 27062226

[cogs70215-bib-0075] Rabi, R. , Miles, S. J. , & Minda, J. P. (2015). Learning categories via rules and similarity: Comparing adults and children. Journal of Experimental Child Psychology, 131, 149–169. 10.1016/j.jecp.2014.10.007 25558860

[cogs70215-bib-0076] Ramachandran, V. S. , & Hubbard, E. M. (2001). Synaesthesia—A window into perception, thought and language. Journal of Consiousness Studies, 8, 3–34.

[cogs70215-bib-0077] Re, D. E. , O'Connor, J. J. , Bennett, P. J. , & Feinberg, D. R. (2012). Preferences for very low and very high voice pitch in humans. PloS One, 7(3), e32719. 10.1371/journal.pone.0032719 22403701 PMC3293852

[cogs70215-bib-0079] Rummer, R. , & Schweppe, J. (2019). Talking emotions: Vowel selection in fictional names depends on the emotional valence of the to‐be‐named faces and objects. Cognition and Emotion, 33(3), 404–416. 10.1080/02699931.2018.1456406 29658373

[cogs70215-bib-0080] Rummer, R. , Schweppe, J. , Schlegelmilch, R. , & Grice, M. (2014). Mood is linked to vowel type: The role of articulatory movements. Emotion, 14, 246–250. 10.1037/a0035752 24708505

[cogs70215-bib-0081] Schmidtke, D. , Körner, A. , Glim, S. , & Rummer, R. (2025). Valence sound symbolism facilitates classification of vowels and emotional facial expressions. Journal of Experimental Psychology: Learning, Memory, and Cognition, 51(4), 661–675. 10.1037/xlm0001389 39325401

[cogs70215-bib-0083] Sidequersky, F. V. , Mapelli, A. , Annoni, I. , Zago, M. , De Felício, C. M. , & Sforza, C. (2016). Three‐dimensional motion analysis of facial movement during verbal and nonverbal expressions in healthy subjects. Clinical Anatomy, 29(8), 991–997. 10.1002/ca.22790 27598053

[cogs70215-bib-0084] Sidhu, D. M. , & Pexman, P. M. (2018). Five mechanisms of sound symbolic association. Psychonomic Bulletin & Review, 25(5), 1619–1643. 10.3758/s13423-017-1361-1 28840520

[cogs70215-bib-0085] Sidhu, D. M. , Athanasopoulou, A. , Archer, S. L. , Czarnecki, N. , Curtin, S. , & Pexman, P. M. (2023). The maluma/takete effect is late: No longitudinal evidence for shape–sound symbolism in the first year. PloS ONE, 18(11), e0287831. 10.1371/journal.pone.0287831 37943758 PMC10635456

[cogs70215-bib-0086] Sidhu, D. M. , Williamson, J. , Slavova, V. , & Pexman, P. M. (2022). An investigation of iconic language development in four datasets. Journal of Child Language, 49(2), 382–396. 10.1017/S0305000921000040 34176538

[cogs70215-bib-0087] Snijders, T. A. B. , & Bosker, R. J. (2012). Multilevel analysis: An introduction to basic and advanced multilevel modeling (2nd ed.). Los Angeles, CA: Sage.

[cogs70215-bib-0089] Stone, G. O. , Vanhoy, M. , & Van Orden, G. C. (1997). Perception is a two‐way street: Feedforward and feedback phonology in visual word recognition. Journal of Memory and Language, 36(3), 337–359. 10.1006/jmla.1996.2487

[cogs70215-bib-0090] Suanda, S. H. , Mugwanya, N. , & Namy, L. L. (2014). Cross‐situational statistical word learning in young children. Journal of Experimental Child Psychology, 126, 395–411. 10.1016/j.jecp.2014.06.003 25015421 PMC4116143

[cogs70215-bib-0092] Taylor, I. K. , & Taylor, M. M. (1965). Another look at phonetic symbolism. Psychological Bulletin, 64(6), 413–427. 10.1037/h0022737 4159231

[cogs70215-bib-0906] Takano, S. , & Honda, K. (2007). An MRI analysis of the extrinsic tongue muscles during vowel production. Speech Communication, 49(1), 49–58. 10.1016/j.specom.2006.09.004

[cogs70215-bib-0093] Theurel, A. , Witt, A. , Malsert, J. , Lejeune, F. , Fiorentini, C. , Barisnikov, K. , & Gentaz, E. (2016). The integration of visual context information in facial emotion recognition in 5‐to 15‐year‐olds. Journal of Experimental Child Psychology, 150, 252–271. 10.1016/j.jecp.2016.06.004 27367301

[cogs70215-bib-0094] Thomas, K. M. , Hunt, R. H. , Vizueta, N. , Sommer, T. , Durston, S. , Yang, Y. , & Worden, M. S. (2004). Evidence of developmental differences in implicit sequence learning: An fMRI study of children and adults. Journal of Cognitive Neuroscience, 16(8), 1339–1351. 10.1162/0898929042304688 15509382

[cogs70215-bib-0095] Thompson, R. L. , Vinson, D. P. , Woll, B. , & Vigliocco, G. (2012). The road to language learning is iconic: Evidence from British sign language. Psychological Science, 23(12), 1443–1448. 10.1177/0956797612459763 23150275

[cogs70215-bib-0096] Tomasello, M. (1992). The social bases of language acquisition. Social Development, 1(1), 67–87. 10.1111/j.1467-9507.1992.tb00135.x

[cogs70215-bib-0097] Trainor, L. J. , & Zacharias, C. A. (1998). Infants prefer higher‐pitched singing. Infant Behavior and Development, 21(4), 799–805. 10.1016/S0163-6383(98)90047-9

[cogs70215-bib-0098] Trainor, L. J. , Austin, C. M. , & Desjardins, R. N. (2000). Is infant‐directed speech prosody a result of the vocal expression of emotion? Psychological Science, 11(3), 188–195. https://doi‐org.ezproxy1.lib.asu.edu/10.1111/1467‐9280.00240 11273402 10.1111/1467-9280.00240

[cogs70215-bib-0099] Tzeng, C. Y. , Nygaard, L. C. , & Namy, L. L. (2017). Developmental change in children's sensitivity to sound symbolism. Journal of Experimental Child Psychology, 160, 107‐118. 10.1016/j.jecp.2017.03.004 28433821

[cogs70215-bib-0100] Vigliocco, G. , Meteyard, L. , Andrews, M. , & Kousta, S. (2009). Toward a theory of semantic representation. Language and Cognition, 1(2), 219–247. 10.1515/LANGCOG.2009.011

[cogs70215-bib-0101] Vogan, V. M. , Morgan, B. R. , Powell, T. L. , Smith, M. L. , & Taylor, M. J. (2016). The neurodevelopmental differences of increasing verbal working memory demand in children and adults. Developmental Cognitive Neuroscience, 17, 19–27. 10.1016/j.dcn.2015.10.008 26615571 PMC6990091

[cogs70215-bib-0102] Walker‐Andrews, A. S. (1998). Emotions and social development: Infants’ recognition of emotions in others. *Pediatrics*, 102(5 Suppl E1), 1268–1271. 10.1542/peds.102.SE1.1268 9794967

[cogs70215-bib-0103] Wang, F. , & Yiu, E. M. ‐L. (2023). Surface electromyographic (sEMG) activity of the suprahyoid and sternocleidomastoid muscles in pitch and loudness control. Frontiers in Physiology, 14, 1147795. 10.3389/fphys.2023.1147795 37215173 PMC10194839

[cogs70215-bib-0104] Warriner, A. B. , Kuperman, V. , & Brysbaert, M. (2013). Norms of valence, arousal, and dominance for 13,915 English lemmas. Behavior Research Methods, 45(4), 1191–1207. 10.3758/s13428-012-0314-x 23404613

[cogs70215-bib-0105] Winter, B. , & Perlman, M. (2021). Size sound symbolism in the English lexicon. Glossa: A Journal of General Linguistics, 6(1). 10.5334/gjgl.1646

[cogs70215-bib-0106] Winter, B. , Sóskuthy, M. , Perlman, M. , & Dingemanse, M. (2022). Trilled /r/is associated with roughness, linking sound and touch across spoken languages. Scientific Reports, 12(1), 1025. 10.1038/s41598-021-04311-7 35058475 PMC8776840

[cogs70215-bib-0107] Yoshida, H. (2012). A cross‐linguistic study of sound symbolism in children's verb learning. Journal of Cognition and Development, 13(2), 232–265. 10.1080/15248372.2011.573515 23807870 PMC3691963

[cogs70215-bib-0108] Yu, C. , & Smith, L. B. (2007). Rapid word learning under uncertainty via cross‐situational statistics. Psychological Science, 18(5), 414–420. 10.1111/j.1467-9280.2007.01915.x 17576281

[cogs70215-bib-0109] Yu, S. P. (2021). *The gleam‐glum effect with pseudo‐words:/i/vs/λ/phonemes carry emotional valence that influences semantic interpretation* (Master's thesis, Arizona State University). KEEP. https://keep.lib.asu.edu/items/161279

[cogs70215-bib-0110] Yu, C. S. P. , McBeath, M. K. , & Glenberg, A. M. (2021a). The gleam‐glum effect: /i:/versus /Ʌ/ phonemes generically carry emotional valence. Journal of Experimental Psychology: Learning, Memory, & Cognition, 47(7), 1173–1185. 10.1037/xlm0001017 34694842

[cogs70215-bib-0111] Yu, C. S. P. , McBeath, M. K. , & Glenberg, A. M. (2021b). Phonemes convey embodied emotion. In M. D. Robinson & L. E. Thomas (Eds.), Handbook of embodied psychology: Thinking, feeling, and acting (pp. 221–243). Cham: Springer International Publishing. 10.1007/978-3-030-78471-3_10

[cogs70215-bib-0112] Ziegler, J. C. , Stone, G. O. , & Jacobs, A. M. (1997). What is the pronunciation for ‐*ough* and the spelling for/u/? A database for computing feedforward and feedback consistency in English. Behavior Research Methods, Instruments, & Computers, 29(4), 600–618. 10.3758/BF03210615

