# Supplementary material for: U.S. English‐Speaking Children and Adults Exhibit a “Gleam‐Glum” Sound Symbolic Effect Linking Phonemic Vowel Sounds With Emotional Valence
Source: Cogn Sci. 2026 Apr 29;50:e70215. doi: 10.1111/cogs.70215 (PMC13128154; doi:10.1111/cogs.70215)
Supplement: Supplementary file 1 — Supporting Information [file COGS-50-e70215-s001.docx]

Supplementary Materials

I Pre-registered data analysis

In our pre-registered plan, we indicated we would conduct t-tests to examine the gleam-glum effect among adults and children, as well as the comparisons of the effect sizes between groups. However, in our manuscript, we opted to conduct generalized linear mixed effects models (GLMM), as this is a more conservative approach and consistent with current trends in psychology research. Here, we present the results of the pre-registered plan, which was consistent with our GLMM results.

To investigate our first aim, whether children would match the pseudowords to emotional pictures consistent with the gleam-glum effect more often than chance (µ_children_ > 0.5), we conducted a one-sample t-test to compare the mean accuracy of children against chance. The mean accuracy was calculated by averaging across scores of the 32 individual test trials (1 as choice matched with the gleam-glum effect, and 0 as choice unmatched with the gleam-glum effect) for each participant. Results showed that the mean accuracy was significantly above chance for children (*M* = .60, *SD* = .15, *p* < .001, *d* = .75), which suggested that children showed a robust gleam-glum effect.

In addition, we assessed whether adults would match the pseudowords to emotional pictures consistent with the gleam-glum effect more often than chance (µ_adults_ > 0.5). We conducted a one-sample t-test to compare the mean accuracy of adults against chance. Results showed that the mean accuracy was significantly above chance for adults (*M* = .75, *SD* = .21, *p* < .001, *d* = 1.18), which suggested that adults showed a robust gleam-glum effect.

To test our second aim, whether children differed from adults in matching pseudowords to emotional pictures consistent with the gleam-glum effect (µ_children_ ≠ µ_adults_), we conducted an independent sample t-test to compare the mean accuracy of adults to that of children. Results showed that adults had a higher mean accuracy than children (*ΔM* = .15, *SD* =.25, *p* < .001, *d* = .60), which suggested that the gleam-glum effect, though being present among both adults and children, was more robust for adults than that for children.

II Adding children’s age as a co-variate in a GLMM to assess the gleam-glum effect

The data analysis below assessed the effect of children’s age on the gleam-glum effect in a GLMM in accordance with reviewers’ comments and was not pre-registered. This accompanies the Results section in the main manuscript, next to the correlational analysis on the link between children’s age and the gleam-glum effect.

To assess the relation between children’s age and the magnitude of the gleam-glum effect, we additionally conducted a GLMM analysis by adding children’s age as a co-variate. This approach was more conservative considering the variation within participants and the variation within tested items, as suggested by the reviewers. The model specification was as follows: score for children ~ children’s age + (1 | Participant) + (1 | Item). Results showed that children’s age did not play a significant role in the effect (*b* = -0.11, *STE* = .11, *z* = -1.02, *p* = .308, 95% CI = [-0.33, 0.11], Odds Ratio = 1.12), consistent with the findings reported in the correlation analysis. In addition, the gleam-glum effect remained significant (*b* = .45, *STE* = .10, *z* = 4.40, *p* < .001, 95% CI = [0.25, 0.66], Odds Ratio = 1.57), confirming the robustness of the effect after controlling for the age differences among young children.

III The effect of alignment between visual images and aurally presented words

The following data analysis accompanies Footnote 4 in the Results section of the manuscript. The data analysis derived from the request of a reviewer and was not pre-registered.

We additionally explored whether the alignment between the visually presented images and the aurally presented words mattered to the gleam-glum effect. In particular, we directly compared the gleam-glum effect for the *Matching trials* when the order of the images matched with the order of the aurally presented words (e.g., the happy image was at the left and the sad image was at the right, at the same time the [i]-pseudoword was presented first and the [Ʌ]-pseudoword was presented second) and for the *Mismatching trials* when the orders of the images and aurally presented words did not match.

We first assessed whether the gleam-glum effect was above chance for the Matching and Mismatching trials respectively. Via GLMM analysis, the gleam-glum effect was significantly above chance for the Matching trials (*b* = 1.88, *STE* = .19, *z* = 9.82, *p* < .0001, 95% CI = [1.53, 2.29], Odds Ratio = 6.56) and for the Mismatching trials (*b* = .96, *STE* = .11, *z* = 8.45, *p* < .0001, 95% CI = [0.74, 1.19], Odds Ratio = 2.62) respectively. We then compared the effects of Matching (Matching versus Mismatching trials) in a GLMM analysis. Results showed that, across participants, the gleam-glum effect was stronger for the Matching trials (*M* = .78, *SD* = .41), compared to the Mismatching trials (*M* =.67, *SD* = .47), *b* = .77, *STE* = 0.12, *z* = 6.65, *p* < .0001, 95% CI = [0.55, 1.02], Odds Ratio = 2.17.

To conclude, the gleam-glum effect was robust no matter how the images and the words were presented spatially-temporally. Yet, the image-word alignment did matter such that the matching trials (where the order of presenting visual images matched with the order of presenting aural words) boosted the gleam-glum effect.

IV The effect of vowel type on the gleam-glum effect

The following data analysis accompanies Footnote 5 in the Results section of the manuscript. The data analysis derived from the request of reviewers and was not pre-registered.

We additionally explored whether the gleam-glum effect differed by vowel type ([i]- versus [Ʌ]-pseudowords) to assess the question regarding whether it was one type vowels, or both vowels, that were driving the gleam-glum effect. In a GLMM, we added trial-by-trial score as the outcome (1, 0), a fixed effect of Vowel type ([i]- versus [Ʌ]-pseudowords, contrast coded), random intercepts for participant and item, and a by-item random slope for Vowel type. When this maximal model did not provide a singular fit, we reduced the model complexity by removing the by-item random slope for Vowel type. The final model specification was as follows: score for all participants ~ Vowel type + (1 | Participant) + (1 | Item). Results showed that the main effect of Vowel type was not significant, suggesting that, across all participants, [i]-pseudowords (*M* = .70, *SD* = .46) and [Ʌ]-pseudowords (*M* = .70, *SD* = .46) contributed to the gleam-glum effect similarly, *b* = 0.05, *STE* = 0.10 *z* = .49, *p* = .625, 95% CI = [-0.16, 0.26], Odds Ratio = 1.05.

To conclude, the exploratory analysis suggests that [i]- and [Ʌ]-pseudowords contributed comparably to the gleam-glum effect. However, we did make it clear that the results from this analysis should be interpreted with caution due to the nature of the methodology as mentioned in the Discussion. That is, we adopted a pseudoword-to-scene mapping paradigm where a participant had to choose one out of two scenes portraiting a positive and a negative scene after hearing a word containing [i] or [Ʌ] vowel. In this case, the link between phoeneme and emotion was always made in comparison, rather than individually. To effectively tease apart which phenoeme(s) drive the effect, future work can adopt a different methdology than the current one, such as increasing the number of candidate scenes of different valences (e.g., happy, sad, calm, exciting, neutral) or having participants rate words individually (rather than in word pairs).
